# The Role of Artificial Neural Networks in Prostate Magnetic Resonance Imaging (MRI) Segmentation

**DOI:** 10.7759/cureus.113599

**Published:** 2026-07-29

**Authors:** Puranjay Shori, Juan Varela, Shreya Shah, Manish Amin, Jong Kim, Victoria Bird

**Affiliations:** 1 Medicine, University of Texas (UT) Southwestern Medical School, Dallas, USA; 2 Urology, University of Florida, Gainesville, USA; 3 Biology and Psychology, University of Florida, Gainesville, USA; 4 MRI Physics, Cortechs.ai, Orlando, USA; 5 Radiology, HCA Florida North Florida Hospital, Gainesville, USA; 6 Urology, National Medical Association and Research Group, Gainesville, USA; 7 Urology, HCA Florida North Florida Hospital, Gainesville, USA

**Keywords:** artificial intelligence, prostate cancer, prostate cancer diagnosis, prostate cancer treatment, prostate segmentation

## Abstract

Introduction

In recent years, artificial intelligence (AI)-generated prostate whole-gland segmentation has shown promise for clinical use. This study compares AI- and human-derived prostate segmentation on magnetic resonance imaging (MRI) and evaluates its practical application.

Methods

A retrospective study from December 2020 to December 2022 evaluated 31 randomly selected patients who had previously undergone MRI-ultrasound (US)-guided fusion biopsies. AI-generated auto-contours of the whole-gland prostate, seminal vesicle, and urethra were produced using the ProtégéAI feature of the MIM Software from MRIs obtained on GE Signa HDxt 3.0T and Siemens Altea Magnetom 1.5T scanners. The same MRIs were independently contoured manually by a board-certified urologist (U) and a board-certified radiologist (R) using MIM Software contouring tools. Volumetric conformity among the three contour sets was assessed using the Dice similarity coefficient, Hausdorff distance (HD), and mean distance to agreement (MDA). Contouring time was recorded for each method, with AI timed from command execution to file generation and physicians timed from file opening to save. Pairwise Wilcoxon signed-rank tests (JMP Pro 15) compared contouring times and similarities across AI to urologist (AI-U), AI to radiologist (AI-R), and urologist to radiologist (U-R) contours.

Results

The average volumetric Dice similarity, HD, and MDA for the AI-U contours were 0.875±0.039, 9.186±4.004 mm, and 1.410±0.511 mm for the whole-gland prostate, 0.283±0.185, 18.117±13.265 mm, and 4.907±4.992 mm for the urethra, and 0.377±0.244, 14.708±9.489 mm, and 4.260±4.092 mm for the seminal vesicle. For the AI-R contours, the values were 0.757±0.072, 17.562±7.240 mm, and 3.050±1.339 mm for the prostate, 0.162±0.105, 19.956±11.962 mm, and 4.798±3.879 mm for the urethra, and 0.451±0.219, 16.069±9.245 mm, and 4.075±3.734 mm for the seminal vesicle. For the U-R contours, the values were 0.769±0.070, 15.109±6.177 mm, and 2.733±1.265 mm for the prostate, 0.144±0.091, 13.560±7.087 mm, and 3.406±1.985 mm for the urethra, and 0.471±0.216, 12.981±6.756 mm, and 3.264±2.308 mm for the seminal vesicle.

Using the Wilcoxon signed-rank test with statistical significance defined as p<0.01, the AI-U Dice similarity for the prostate differed significantly from both the AI-R and U-R comparisons. Similarly, AI-U demonstrated significantly different HD values than both AI-R and U-R, whereas the AI-R and U-R HD comparison was statistically insignificant. All pairwise MDA comparisons for the prostate were statistically significant. For the urethra, the AI-U Dice similarity differed from the other two comparisons, while for HD, the AI-R and U-R comparisons differed significantly from each other. No significant differences were observed among the three comparisons for the seminal vesicle across any metric. The average times to produce contours for the AI, urologist, and radiologist were 96.5 seconds, 285.8 seconds, and 217.9 seconds, respectively (p<0.01 for each time comparison).

Conclusion

This study suggests that AI may be a useful tool for prostate segmentation workflows in streamlining MRI-US prostate cancer diagnostics by producing similar contouring results in less time. Additional investigation should be conducted regarding the differences between the pathological outcomes of AI and non-AI contours, and the accuracy, cost analysis, and efficiency of AI technology should be elucidated.

## Introduction

Prostate cancer remains the leading non-skin cancer diagnosis among men in the United States, with over 300,000 new cases and over 35,000 deaths projected in 2026 [1-3]. Additionally, it ranked as the sixth leading cause of cancer-related deaths globally [4]. This highlights the importance of timely prostate cancer screening.

The standard of care for prostate cancer diagnosis following screening is a prostate biopsy (PBx), through either the transrectal or the transperineal route [5]. Approximately one million PBxs are performed in the United States annually, and recent data suggest that close to 40% of all biopsies will be positive [5,6].

However, the field of urology has seen a mismatch between the availability of physicians and their increasing demand, an issue that artificial intelligence (AI) could prove crucial in bridging this gap [1,7].

In recent years, magnetic resonance imaging (MRI) has been used in conjunction with ultrasound (US) technology to conduct biopsies of the prostate through MRI-US-guided fusion biopsy [8]. The success of MRI-US-guided fusion biopsies depends on the radiologist's level of urologic experience, involving extensive training and accurate segmentation of the prostate, either adjacent to or within the organ [9]. 

One of the clinical applications of AI has been its ability to aid in medical imaging, particularly the contouring of structures such as organs and lesions [10]. Within urology, the promising union between AI contouring and image-guided biopsies may aid the timely and effective diagnosis of prostate cancer. Within this study, an AI model, the MIM Software "ProtégéAI" (Beachwood, Ohio, United States), was tested against the standard of care for segmenting prostate MRIs.

The MIM Software is a tool used to view and contour radiological scans. ProtégéAI+™ is a MIM Software tool that received its initial US Food and Drug Administration (FDA) clearance on February 3, 2022, under 510(k) number K213976. ProtégéAI utilizes a subset of AI called a neural network (NN) based on U-Net, artificial NN, and convolutional NN architecture to contour MRIs [11]. Belonging to the narrow AI application with limited memory functionality, this tool is specifically tailored to automate and standardize the contouring process in organ segmentation. ProtégéAI uniquely enables physicians to use commercial AI software for organ segmentation, requiring only a connection to the MIM Software cloud server to upload MR views and receive contoured images. 

This study aimed to compare AI-generated contours of the prostate, seminal vesicles, and urethra on prostate MRI with physician-generated contours created by a board-certified urologist and radiologist. Contour agreement was evaluated using the Dice similarity coefficient, Hausdorff distance (HD), mean distance to agreement (MDA), and contouring time. We hypothesized that ProtégéAI would demonstrate contour agreement comparable to physician-generated contours while significantly reducing contouring time.

## Materials and methods

A retrospective study was conducted from December 2020 to December 2022 at Urologic Integrated Care in Gainesville, Florida. AI-generated auto-contours for prostate MRIs of patients who had previously undergone MRI-US-guided fusion biopsies at this institution were procured through the ProtégéAI feature of the MIM Software to segment the prostate, seminal vesicles, and urethra. ProtégéAI was used in its standard configuration throughout the study, and the underlying static AI model was not retrained or adapted using study images or physician-generated contours.

Eligible cases included patients with complete MRI datasets containing axial, sagittal, and coronal T2-weighted images. Axial T2-weighted images were required because they served as the input for ProtégéAI contour generation, whereas sagittal and coronal images were required to allow manual contouring and review by both the board-certified urologist and board-certified radiologist. MRI examinations lacking any of these required image sequences were excluded. MRIs were obtained using the GE Signa HDxt 3.0T scanner (GE HealthCare, Chicago, Illinois, United States) and the Siemens Altea Magnetom 1.5T scanner (Siemens Healthineers, Erlangen, Germany) at an outpatient imaging facility. The Siemens Altea Magnetom 1.5T scanner had a gradient amplitude of 33 milliTesla/meter for each gradient axis. Diffusion-weighted imaging (DWI) b-values included 50, 100, 300, 800, and 1000, with an auto-generated 1400. 

Following the application of the inclusion and exclusion criteria, 31 patients were randomly selected from eligible MRI examinations using a computer-generated randomization procedure. Because this was an exploratory retrospective study, the sample size was determined by the resource-intensive process of expert manual contour generation, contour comparison, and data abstraction and the limited availability of eligible MRI examinations. MRIs were also contoured manually by both a board-certified urologist and a board-certified radiologist using contouring tools found in the MIM Software, which marked individual slices of the MRI and connected them to provide a 3-D contour. Manual contouring was performed according to each physician's standard clinical practice without additional study-specific contouring guidelines. Manual contours were generated independently by the board-certified urologist and board-certified radiologist, each blinded to the AI-generated contours and to the other physician's contours. AI-generated contours were analyzed without manual modification or post-processing. The volumetric conformity of the three contour sets was evaluated by calculating the Dice coefficients, HD, and MDA. All contour comparisons were performed using the same MRI dataset for each patient, with axial T2-weighted images used for contour generation and sagittal and coronal T2-weighted images used for reference during manual contouring.

The Dice coefficient is a metric utilized in medical imaging to determine the spatial overlap of two tracings [12]. The MDA is the average of all of the distances to agreements or the shortest distance from a point on one contour to another on the second contour [12]. The HD is defined as the largest distance of a point in a set to the closest point in the other set.

The time required to generate or manually create contours was recorded. To measure contouring time in a manner reflecting routine clinical workflow, the AI contours were timed from the moment the human operator executed the command to generate the contour to the completion of the contour and generation of a usable file. To measure the time elapsed for the urologist and radiologist, both physicians were timed from when they opened the necessary files to begin a contour to the moment of saving their produced contours.

The similarities of the Dice coefficient, HD, and MDA for the prostate, seminal vesicles, and urethra between the AI and urologist (AI-U), AI and radiologist (AI-R), and radiologist and urologist (U-R) were determined through pairwise Wilcoxon signed-rank tests. The times to produce contours for each different method of generating contours were also compared using pairwise Wilcoxon signed-rank tests. Statistical analysis was completed using JMP Pro 15 (SAS Institute Inc., Cary, North Carolina, United States).

## Results

For the prostate, the average Dice similarity, HD, and MDA for the prostate contours were 87.50%, 9.186 mm, and 1.41 mm for the AI-U comparison, 75.70%, 17.562 mm, and 3.050 mm for the AI-R comparison, and 76.90%, 15.109 mm, and 2.733 mm for the U-R comparison. The difference in Dice similarity between the AI-R and U-R contours was statistically insignificant for the prostate, though both of these pairs differed significantly from the AI-U comparison (p<0.01). Furthermore, for prostate HD, AI-U differed significantly (p<0.01) from both AI-R and U-R, whereas the AI-R versus U-R comparison was not statistically significant. Each of the MDA comparisons for AI-U, AI-R, and U-R proved to be significantly different from one another (p<0.01) (Table 1). 

**Table 1:** Dice similarity, HD, and MDA between AI-, urologist-, and radiologist-generated contours for the prostate, urethra, and seminal vesicles (n=31) Values are presented as mean±standard deviation. HD: Hausdorff distance; MDA: mean distance to agreement; AI: artificial intelligence; AI-U: AI to urologist; AI-R: AI to radiologist; U-R: urologist to radiologist

Organ	Comparison	Dice similarity	HD (mm)	MDA (mm)
Prostate	AI-U	0.875±0.039	9.186±4.004	1.410±0.511
	AI-R	0.757±0.072	17.562±7.240	3.050±1.339
	U-R	0.769±0.070	15.109±6.177	2.733±1.265
Urethra	AI-U	0.283±0.185	18.117±13.265	4.907±4.992
	AI-R	0.162±0.105	19.956±11.962	4.798±3.879
	U-R	0.144±0.091	13.560±7.087	3.406±1.985
Seminal vesicle	AI-U	0.377±0.244	14.708±9.489	4.260±4.092
	AI-R	0.451±0.219	16.069±9.245	4.075±3.734
	U-R	0.471±0.216	12.981±6.756	3.264±2.308

**Figure 1:**
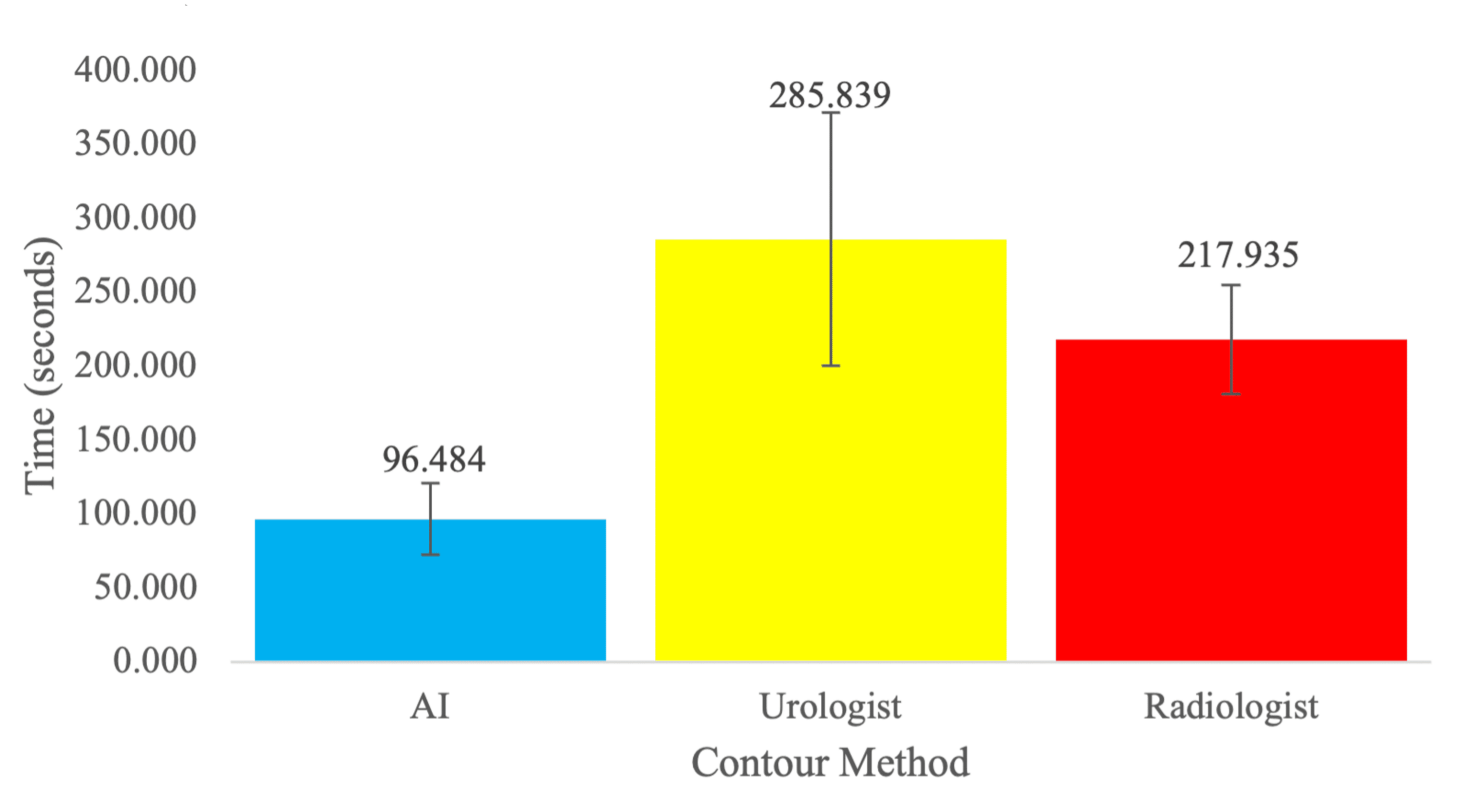
Average contouring time (seconds) for AI, urologist, and radiologist contour generation Error bars represent ±1 standard deviation. Pairwise comparisons were performed using two-sided Wilcoxon signed-rank tests (AI vs. urologist: S=248.0; p<0.0001; AI vs. radiologist: S=248.0; p<0.0001; urologist vs. radiologist: S=-206.5; p<0.0001). AI: artificial intelligence

For the urethra, the average Dice similarity, HD, and MDA for the urethral contours were 28.30%, 18.117 mm, and 4.907 mm for the AI-U comparison, 16.20%, 19.956 mm, and 4.798 mm for the AI-R comparison, and 14.40%, 13.560 mm, and 3.406 mm for the U-R comparison. A similar statistical trend to the prostate Dice similarity was observed. However, no significant differences were observed for the urethral MDA (p>0.01), and the urethral HDs of AI-R and U-R were found to be significantly different from each other (p<0.01), while both still maintained a statistically similar profile to the AI-U comparison (p>0.01), as illustrated in Figure 1. In contrast to the variations observed in the prostate and urethra, no statistical differences were observed for the seminal vesicle when comparing the three observer groups across any of the evaluated metrics (p>0.01). The average Dice similarity, HD, and MDA were 37.7%, 14.708 mm, and 4.260 mm for the AI-U comparison, 45.1%, 16.069 mm, and 4.075 mm for the AI-R comparison, and 47.1%, 12.981 mm, and 3.264 mm for the U-R comparison, respectively. This may indicate either greater baseline agreement or uniformly high variability among contours for the seminal vesicle, failing to reach the threshold of statistical significance across the tested parameters.

When evaluating procedural efficiency, the average times required to produce contours for the AI, the urologist, and the radiologist were 96.5 seconds, 285.8 seconds, and 217.9 seconds, respectively. These differences represented a highly significant time savings and variance between each group (p<0.01 calculated via the Wilcoxon signed-rank test). Because the Dice similarity coefficient reflects spatial overlap, these findings indicate unequal degrees of geometric agreement among observer comparisons (Table 2).

**Table 2:** Pairwise Wilcoxon signed-rank test results for Dice similarity, HD, and MDA (n=31) Two-sided p-values are reported. Statistical significance (*) was defined as p<0.01. HD: Hausdorff distance; MDA: mean distance to agreement; AI: artificial intelligence; AI-U: AI to urologist; AI-R: AI to radiologist; U-R: urologist to radiologist

Organ	Pairwise comparison	Wilcoxon S	P-value
Dice similarity			
Prostate	AI-U vs. AI-R	-248	<0.0001*
	AI-U vs. U-R	-248	<0.0001*
	AI-R vs. U-R	102	0.0436
Urethra	AI-U vs. AI-R	-146.5	0.0025*
	AI-U vs. U-R	-189.5	<0.0001*
	AI-R vs. U-R	-40	0.4422
Seminal vesicle	AI-U vs. AI-R	94.5	0.0628
	AI-U vs. U-R	78	0.1284
	AI-R vs. U-R	16	0.7595
HD			
Prostate	AI-U vs. AI-R	247	<0.0001*
	AI-U vs. U-R	211	<0.0001*
	AI-R vs. U-R	-116.5	0.0194
Urethra	AI-U vs. AI-R	68	0.1871
	AI-U vs. U-R	-66	0.2008
	AI-R vs. U-R	-151	0.0017*
Seminal vesicle	AI-U vs. AI-R	88	0.0845
	AI-U vs. U-R	-25	0.632
	AI-R vs. U-R	-81	0.1137
MDA			
Prostate	AI-U vs. AI-R	248	<0.0001*
	AI-U vs. U-R	237	<0.0001*
	AI-R vs. U-R	-149	0.0021*
Urethra	AI-U vs. AI-R	21	0.6877
	AI-U vs. U-R	-34	0.5142
	AI-R vs. U-R	-100	0.0482
Seminal vesicle	AI-U vs. AI-R	-6	0.9087
	AI-U vs. U-R	-6	0.9087
	AI-R vs. U-R	-44	0.3974

**Figure 2:**
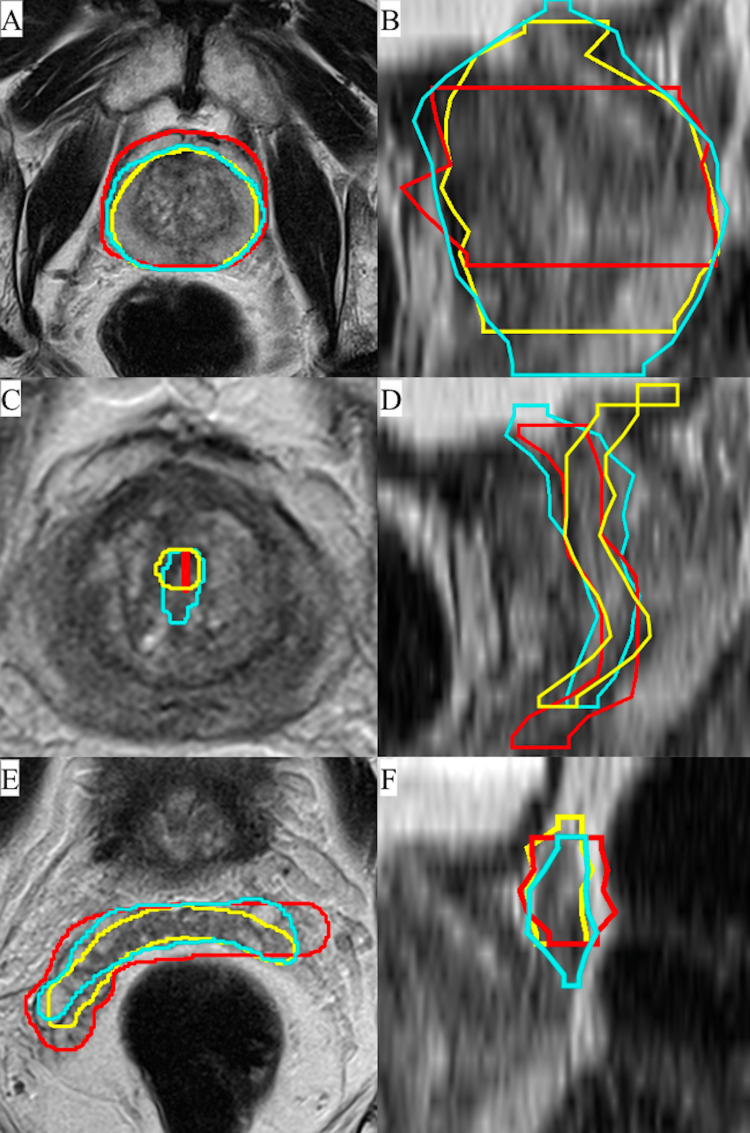
Representative T2-weighted prostate MRIs demonstrating AI-, urologist-, and radiologist-generated contours: (A) axial view of the prostate, (B) sagittal view of the prostate, (C) axial view of the urethra, (D) sagittal view of the urethra, (E) axial view of the seminal vesicles, and (F) sagittal view of the seminal vesicles AI-generated contours are shown in cyan, urologist contours in yellow, and radiologist contours in red. AI: artificial intelligence; MRI: magnetic resonance imaging

Figure 2 depicts representative T2-weighted prostate MRIs demonstrating AI-, urologist-, and radiologist-generated contours.

## Discussion

Imaging guidance through MRI targeting has played a prominent role in the improved efficacy of PBxs in diagnosing clinically significant prostate cancer and reducing costs through the decreased detection of indolent cancers [9]. The expanded adoption of MRI scans and biomarkers in identifying prostate cancer holds promise for minimizing the adverse outcomes linked with screening efforts [13].

Despite its advantages, the use of MRI in prostate cancer diagnosis can be time-consuming, which is problematic if interpretation of imaging is necessary before a PBx [14]. This can pose challenges for urologists who are already skeptical about the increased implementation of MRI fusion biopsies [15]. AI has emerged as a tool for solving these limitations through AI-driven medical imaging interpretation and diagnostic assistance to help providers make faster and more accurate diagnoses, predict disease progression, and recommend personalized treatment plans [16].

To understand the role of AI in diagnosing prostate cancer, there must be an evaluation of the current limitations within the current PBx practices in urology for diagnosing patients with an elevated prostate-specific antigen (PSA) and associated prostate cancer biomarkers. Patients being evaluated for prostate cancer face challenges with costs that vary depending on the use of imaging modalities such as MRI, the clinical location they visit (outpatient office vs. ambulatory surgical center), the price of anesthesia, and the implementation of a transrectal or transperineal approach [17]. The efficacy and accuracy of PBxs are limited by the availability of these tools and their clinical setting, often coming down to patient choice based on affordability. Costs likewise extend past the date of PBx, as Weiner et al. have demonstrated: 46% of biopsy costs will arrive 30 days following the procedure, involving further hospitalization due to rare complications like urosepsis and severe rectal bleeding, in contrast to the more common hematuria, hematospermia, and rectal bleeding [17,18]. Additionally, there is emerging evidence that PBxs are likewise limited in detecting cribriform and intraductal prostate cancers, emphasizing the need for improved biomarkers [19].

AI-generated contours of organs and whole-gland segmentation have shown potential for application as a time-saving and accurate tool in the clinical setting [20]. A study conducted by Hindocha et al. found that almost half of the oncologists in the Royal College of Radiologists were using AI in their departments for organ contouring, with the most reported organs and tumor sites being the brain, head and neck, thorax, abdomen, prostate, bladder, and skin [20]. The use of AI for organ segmentation has grown, reflected by the increasing number of robust studies in the available literature. ProtégéAI has shown efficacy comparable to manual contouring in CT analyses of similar cohort sizes, as Goddard et al. demonstrated in their delineation of 16 different organs at risk (OARs) for radiation therapy [21]. Similarly, ProtégéAI fared equally to other deep learning-based auto-segmentation (DLAS) tools and manual contouring in a study by Johnson et al., in which 40 clinical head and neck cancer (HNC) cases were auto-contoured on planning computed tomography (pCT) [22]. To our knowledge, our study is the most extensive in assessing the ability of ProtégéAI to contour regions of interest (ROIs) on MRI scans for the diagnosis and treatment of prostate cancer.

This study suggests that ProtégéAI contours of the prostate show reasonable volumetric conformity with manual contouring by a urologic surgeon based on the Dice coefficient. When compared with other literature investigating the Dice similarity of prostate segmentation completed by AI and manually, Wang et al. report a Dice coefficient of 0.86 and an HD of 7.98 mm for the prostate, which are similar to the results for comparisons of the prostate found in this study [23]. Additional studies, like those of Zou et al., also suggested similar Dice similarity with a mean Dice similarity of 0.883 for comparisons of manual and AI segmentation of the peripheral zone of the prostate on MRIs [24]. Duan et al. report a Dice similarity of 0.72 for the seminal vesicles and a Dice similarity of 0.83 and an HD of 6.07 for the prostate on computed tomography (CT) [25]. These comparisons to other studies suggest that, especially in terms of Dice similarity, the similarity of the AI used in this study was of an acceptable and commonly observed conformity with manual contours for the prostate.

The lack of literature on comparisons of AI contours to manual contouring of the urethra also makes it unknown whether a higher similarity is expected for the urethra and whether the HD and MDA are also to be expected. Because the Dice coefficient is dependent on the volume of the structure, a smaller structure, such as the urethra, may have different conformities than expected [26]. We believe the learning curve is steeper for urethral segmentation than for prostate or seminal vesicles. The seminal vesicle also showed lower similarity than other reports of this organ's Dice similarity and the overall range in which such Dice similarities are considered acceptable. The data gathered for comparing the contours of the seminal vesicle all have higher standard deviations from the mean. This similarity may improve with the addition of a larger sample size.

Outside of the volumetric conformity, the prostate HD results demonstrated significant differences between the AI-U versus AI-R and AI-U versus U-R comparisons, whereas the AI-R versus U-R comparison was not statistically significant. In contrast, all pairwise MDA comparisons were statistically significant, indicating differing degrees of contour shape variation among the observer groups. The variability characterized in part through the MDA was overall lower in the prostate with a range of 1.4-3.1 mm, followed by the seminal vesicle with a range of 3.3-4.3 mm, and higher in the urethra (3.4-4.9 mm). For the urethra, only the AI-R and U-R HD comparison reached statistical significance (p<0.01), whereas neither comparison involving AI-U was statistically different. This finding may reflect variability in physician contouring of the urethra rather than a consistent difference attributable to the AI. Because urethral segmentation is inherently challenging and often requires estimation when the urethra is poorly visualized on MRI, additional studies with larger cohorts are needed to better characterize these differences. MRI fusion biopsy has multiple components that require physicians' time and input. Prostate contouring is one of these items, and AI-driven prostate segmentation in our study was performed more than twice as fast compared to the urologist and radiologist. Shortening input for this component may further aid the urologist in accurately and efficiently performing this procedure.

This study may be limited by the urologist's greater familiarity with the clinical contouring workflow. However, the AI is described as a static model and does not require or utilize user-submitted datasets, limiting the chance of it aligning to the urologist's contours. Because the deployed AI model does not appear to improve from individual user edits, future iterations of the model may benefit from training on larger and more diverse physician-generated contour datasets to improve robustness and generalizability. A related limitation of the study is the difficulty in establishing a consensus reference standard (gold standard) for comparison. As the results suggest, contours between two physicians can vary. Hence, this makes it difficult to make a "true" comparison of a subjective contour and the automated segmentation of anatomical structures on MRI. Thus, further studies that compare the data of the AI contours to several different physicians would assist in validating the trends observed within this study. Another consideration would be to consolidate the contours of several clinicians to create a more accurate "gold standard" to use as a comparison for determining the acceptable volumetric similarity, as is done in several other studies comparing different contours [27,28]. Another limitation of this study is its retrospective, single-institution design and relatively small sample size, which may affect the generalizability of the findings and underscores the need for further research with larger cohorts. The sample size was constrained by the time-intensive process of uploading MRI datasets for AI contour generation, performing expert manual contouring and contour comparisons, and the limited availability of eligible MRI examinations. Additionally, this study evaluated whole-organ segmentation rather than lesion detection, biopsy accuracy, pathological correlation, clinical outcomes, or cost-effectiveness. Therefore, the findings primarily support the feasibility of AI-assisted contouring and workflow efficiency rather than improvements in prostate cancer diagnosis or patient outcomes.

The study aimed to compare the relative differences between auto-contours and human contours. While the comparisons with the physicians did not meet the recommended values for Dice, HD, and MDA, the AI comparisons not meeting these recommended values do not detract from the efficacy of the contours, as the U-R comparisons were similar. Even more, the contour comparison closest to meeting the recommendation for high registration accuracy was the AI-U comparison for the prostate, which suggests high levels of volumetric conformity and lack of shape variation when comparing AI to the contours of urologists. However, given the already mentioned limitations of the study coupled with the small sample size and large standard deviations, these values of the HD and MDA may become more acceptable with additional tested samples and a larger pool of physicians to compare contours with.

This study analyzed the efficacy of AI to contour the prostate, seminal vesicles, and urethra. As suggested by other studies, further testing of AI tools specializing in tumor segmentation is necessary to assist further in prostate cancer biopsies and determine the outcomes of diagnostic tests [29]. Additional developments in AI tools must be made in lesion contouring if AI is to become a vital tool or total replacement for physician interpretation of MRIs. However, because it can be as similar to the contours of two physicians for the prostate, seminal vesicles, and urethra, AI has the potential to be a useful tool to assist physicians in the clinical setting rather than a replacement for human interpretation [30]. In fact, in the clinical setting where patient MRI files were collected, AI-derived auto-contours have been consistently used in conjunction with minor or no revisions by a board-certified urologist to perform fusion biopsies continuously following this initial study in a process known as human-enhanced AI (HEAI).

Incorporating ProtégéAI into clinical workflows should emphasize transparency and responsible application, positioning AI as a supportive tool that enhances radiologists' efficiency without replacing their expertise. Additionally, issues with the liability and accountability of AI will become increasingly significant as its medical applications continue to expand. By keeping clinicians central in interpreting AI-generated contours, facilities can streamline processes while ensuring that patient care remains accurate and tailored to individual anatomical needs.

## Conclusions

The results of this study suggest that AI may serve as a useful tool for streamlining prostate MRI contouring and supporting clinical workflow. In this study, ProtégéAI produced prostate contours with reasonable similarity to physician-generated contours while substantially reducing contouring time compared with both the urologist and radiologist. Given the limited dataset of this study, further studies must be conducted with larger sample sizes and additional contouring physicians to further evaluate the accuracy, generalizability, and clinical utility of AI-assisted contouring.
